# Evaluation of the CENTURY Model Using Long-Term Fertilization Trials under Corn-Wheat Cropping Systems in the Typical Croplands of China

**DOI:** 10.1371/journal.pone.0095142

**Published:** 2014-04-21

**Authors:** Rihuan Cong, Xiujun Wang, Minggang Xu, Stephen M. Ogle, William J. Parton

**Affiliations:** 1 Ministry of Agriculture Key Laboratory of Crop Nutrition and Fertilization, Institute of Agricultural Resources and Regional Planning, Chinese Academy of Agricultural Sciences, Beijing, China; 2 College of Resources and Environment, Huazhong Agricultural University, Wuhan, China; 3 State Key Laboratory of Desert and Oasis Ecology, Xinjiang Institute of Ecology and Geography, Chinese Academy of Sciences, Urumqi, China; 4 Earth System Science Interdisciplinary Center, University of Maryland, College Park, Maryland, United States of America; 5 Natural Resource Ecology Laboratory, Colorado State University, Fort Collins, Colorado, United States of America; University of Erlangen-Nuremberg, Germany

## Abstract

Soil organic matter models are widely used to study soil organic carbon (SOC) dynamics. Here, we used the CENTURY model to simulate SOC in wheat-corn cropping systems at three long-term fertilization trials. Our study indicates that CENTURY can simulate fertilization effects on SOC dynamics under different climate and soil conditions. The normalized root mean square error is less than 15% for all the treatments. Soil carbon presents various changes under different fertilization management. Treatment with straw return would enhance SOC to a relatively stable level whereas chemical fertilization affects SOC differently across the three sites. After running CENTURY over the period of 1990–2050, the SOC levels are predicted to increase from 31.8 to 52.1 Mg ha^−1^ across the three sites. We estimate that the carbon sequestration potential between 1990 and 2050 would be 9.4–35.7 Mg ha^−1^ under the current high manure application at the three sites. Analysis of SOC in each carbon pool indicates that long-term fertilization enhances the slow pool proportion but decreases the passive pool proportion. Model results suggest that change in the slow carbon pool is the major driver of the overall trends in SOC stocks under long-term fertilization.

## Introduction

Soil organic carbon (SOC) is one of the most important terrestrial pools for C storage. It is estimated that the total soil carbon pool is around 1400–1500 Pg C, which is approximately three times greater than the atmospheric pool (750 Pg C) [1], [2]. The SOC pool represents a dynamic equilibrium resulting from changes in gains and losses. Even small changes in SOC at a site may potentially add up to significant changes in large-scale carbon cycling across a region [3]. Furthermore, SOC is relatively dynamic and can be greatly influenced by agricultural practices. Increases in SOC storage in cropland soils would benefit soil productivity and environmental health [4], [5], and so alternative farming management practices have been evaluated to identify their potentials for increasing SOC in the agroecosystems [4]–[7].

Long-term experiments are crucial for determining fundamental crop, soil and ecological processes and their impacts on the environment [6]–[9]. Data from long-term experiments provide a unique resource to investigate long-term influences of climate, crop rotation and crop residue management on soil fertility [6]–[12]. However, SOC change is affected by complex interactions that vary across space and time depending on the environmental conditions and agricultural management practices. A weakness of long-term experiments is that they are typically restricted to small subset of the entire set of environmental conditions and management practices that exists [13].

Process-based models are an effective way to evaluate SOC changes across a broader set of environmental conditions and management practices [14]. In recent decades, the development and evaluation of soil organic matter models has improved the understanding of factors controlling SOC dynamics, and thus increased our ability to predict future SOC trends. A number of SOC models have been developed, but applying these models requires adequate evaluation with measured SOC trends from experimental for different environmental conditions and management practices [15]. For example, the CENTURY model [16] has been widely used to simulate SOC changes under different management conditions in long-term experiments (e.g., [17], [18] and [19]). With the development of CENTURY, the model has been successfully employed in long-term fertilizer, irrigation, pest management, and site-specific farming applications [20], [21]. In China, CENTURY model has been used in grassland [22], forest [23], and regional farmland [24]. However, CENTURY modeling research was still limited in farmland especially under the double cropping rotations and in the acidic soil.

Here, we evaluate the CENTURY with data from three long-term experiments with wheat-corn cropping rotations and different fertilization practices. Specifically, our objectives were (i) to evaluate the performance of CENTURY with evaluation of modeled SOC stocks for different fertilizations and under acidic soil; (ii) to study the effect of fertilization practices on different SOC pools in the modeling framework; and (iii) to predict soil carbon potential under long-term fertilization.

## Materials and Methods

### Long-term Experiment

Three long-term experiments were utilized for this study, which were located at Changping (40°13′N, 116°15′E), Yangling (34°17′N, 108°00′E) and Qiyang (26°45′N, 111°52′E) in China. Climate conditions varied from semi-humid (Changping site) to humid warm-temperate (Yangling sites) to humid subtropical climate (Qiyang site). Annual mean temperature was 13.1°C at the Changping site, 14.9°C at the Yangling site, and 18.1°C at the Qiyang site. Annual precipitation was generally low at Changping (515 mm) and Yangling (525 mm) sites but 1445 mm at Qiyang. However, annual evaporation was much higher, varying from 993 mm to 1470 mm [25].

The experimental sites had double cropping systems, i.e., winter wheat and summer corn. Winter wheat was seeded in early November and harvested in early May at the Qiyang site. For the other two sites, winter wheat was seeded around October 20^th^, and harvested around June 1^st^. The wheat seeding rates ranged from 165 to 225 kg ha^−1^. Summer corn was sown around June 10^th^, with the rate of 63 000–75 000 seeds per hectare, and harvested around October 1^st^ at most sites. For the Qiyang site, summer corn was sown in holes between the wheat strips in early April, and harvested in the middle of July. Thus, there was a short period of inter-cropping for wheat and corn at the Qiyang site. However, in the CENTURY modeling, wheat was harvested in April and corn was sown in May at the Qiyang site since inter-cropping could not be fitted in the model. We believed that there would be little impact on the belowground biomass of wheat and corn, and thus to the soil organic carbon turnover.

Seven treatments were selected in this study: (i) control (no fertilizer); (ii) mineral nitrogen (N); (iii) mineral nitrogen and phosphorus combination (NP); (iv) mineral nitrogen, phosphorus and potassium combination (NPK); (v) NPK combinations with livestock or farmyard (i.e., livestock manure mixed with soil and/or crop residue) manure (NPKM); (vi) 1.5 times’ application rate of NPKM (hNPKM); and (vii) mineral NPK combined with crop straw (NPKS). The total nitrogen applied (i.e., mineral plus organic) was equal (i.e., nitrogen balanced) in each of the fertilizer treatments (i.e., N, NP, NPK, NPKM and NPKS treatments). The NPKM and hNPKM treatments had 30% of total N applied from mineral fertilizer and the remaining 70% from organic manure. The treatment plots were initially randomized and isolated by 100-cm-cement baffle plates. There was no replicate for the treatments at these sites due to field availability. However, the plot size was relative large (196 m^2^–400 m^2^) at each study site. The durations of experiments were from 1990 to 2005 at the Changping site, from 1990 to 2008 at the Qiyang sites, and from 1990 to 2009 at the Yangling site.

Urea, calcium superphosphate, and potassium chloride were used as sources of mineral N, P, and K, respectively. All P and K fertilizers and nearly half of the mineral N fertilizer were applied as basal fertilizers prior to seeding. The remaining mineral N fertilizer was applied as top dressing during the growing season. Manure was generally applied once each year before wheat sowing. However, at the Qiyang site, 30% of manure was applied before wheat seeding and remaining 70% was applied before corn seeding. The sources of organic manure were farmyard manure mixed with crop residue at Changping site, cattle manure at Yangling site, and pig manure at Qiyang site [25]. For the NPKS treatment, half amount (2000 kg ha^−1 ^yr^−1^) of both wheat and corn straw was incorporated at the Qiyang site, whereas 2250 kg ha^−1 ^yr^−1^ and 4500 kg ha^−1 ^yr^−1^ of corn straw were incorporated at the Changping and Yangling sites, respectively [26]. Carbon and nutrient contents of manure are measured in 2009 and 2010. Nutrient contents of straw are measured annually over the period of experiment.

There was no irrigation at the Qiyang site since sufficient precipitation occurs during the growing season (i.e., from March to August). However, at the other two sites, irrigations were applied 2–3 times during wheat growing season: 5 mm before seeding, 4 mm for the over wintering stage if needed, and 5 mm at jointing stage. Irrigation was applied once during corn growing season, with 5 mm before seeding.

Plots were ploughed to a depth of 20 cm twice a year, usually in early June at most sites after wheat harvest and early October after corn harvest. In contrast, at the Qiyang site, the whole plot area was ploughed (20 cm depth) in late October before wheat seeding. Then, area between the wheat belts was ploughed (20 cm depth) in early April before the corn seeding.

Aboveground biomass was removed with negligible stubble left in the field. Thus, organic carbon input was mainly from manure application (i.e., NPKM and hNPKM treatments), residue addition (i.e., NPKS treatment) and from root system during crop growing season. All harvested biomass was removed from the plots for determining crop grain and residue yields. The grain and residue samples were air-dried, threshed, oven-dried at 70°C to a uniform moisture level, and then weighed separately.

During the experimental periods, surface soil (0–20 cm) was collected in each plot annually approximately 15 days after corn harvest. Five cores of soil from each plot were randomly taken, and soils were mixed thoroughly, and air dried for seven days after removing discrete plant residues and visible soil organism. Air-dried soil was sieved through 2 mm screen to determine pH (1∶1w/v water). Representative subsamples were crushed to 0.25 mm for measurements of SOC [27]. Soil bulk density was measured in situ once every five or ten years. Surface soil SOC stock expressed as Mg ha^−1^ was calculated by multiplying the SOC content (g kg^−1^) by averaged soil bulk density (g cm^−3^) and depth (20 cm).

### Model Description and Application

**Figure 1:**
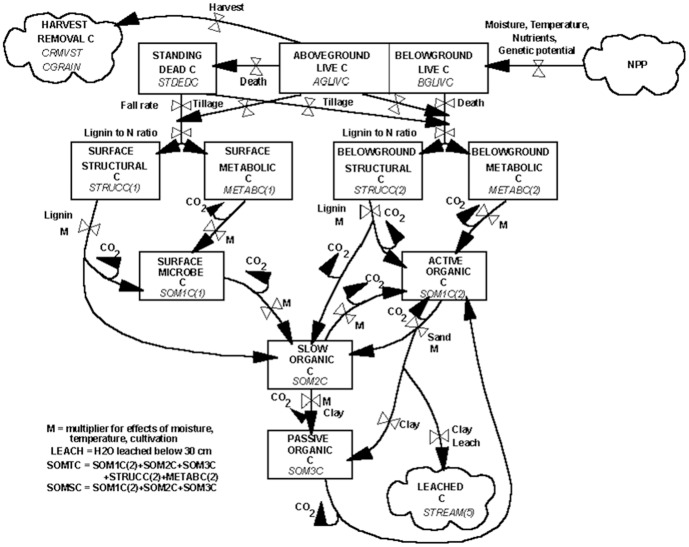
The pools and flows of carbon in the CENTURY model. The diagram showed the major factors which control the flows [29].

The simulation of SOC dynamics was performed with the CENTURY model (version 4.5) which was described by Parton and Rasmussen [28]. As shown in Fig. 1 [29], the arrows showing the CO_2_ evolved in the transformations was indicative of the microbial growth efficiencies. The first-order decay rates for each of the pools corresponded to turnover times of roughly 3 and 0.5 years for the structural and metabolic components; 1.5 years for the active fraction, 25 years for the slow pool, and 1000 years for the passive pool [28]. The actual turnover times of each carbon pool was a function of the maximum turnover time of specific carbon pool and DEFAC. The value of DEFAC was calculated by multiplying the soil moisture factor (function of precipitation and stored soil water) and the soil temperature factor (function of the average monthly soil surface temperature). The turnover rate of active carbon pool was also a function of soil texture (higher for sandy soils), while the stabilization of active carbon into slow carbon was a function of the silt plus clay content [28].

Model simulations were set-up based on the historical crop rotations and farm practices’ investigation from the local farmers at these sites. Five distinct periods were modeled (Table 1), including (1) an initialization period, i.e., 4000 years of native vegetation to reach an equilibrium; (2) the first cultivation period, i.e., plowing of the native grassland in 1901, and planting of corn with removal of straw until 1960; (3) a second cultivation period, i.e., corn-wheat cropping with removal of straw from 1961 to 1988; (4) pre-experimental treatment period, i.e., uniform tillage with no crop in 1989 prior to establishing the long-term experiment; and (5) experiment period, i.e., the long-term corn-wheat fertility experiment from 1990 to 2050. Management during the experiment (1990–2010) was repeated for remainder of the experimental period until the end time of simulation to evaluate future trends. As shown in Table 2, carbon inputs from manure application were around 500 kg C ha^−1^ before the long-term experiment. Then carbon input varied based on measured data at different sites.

**Table 1:** Land management practices for the long-term experimental sites during the different blocks and periods used in the CENTURY model simulations.

No.	Periods	Management practices	Repeating sequence
1	up to 1900 (4000 yrs)	Native grassland, low-intensity grazing	1 year
2	1901–1960	Corn, low crop yield cultivar, ploughing, remove straw, applying manure	1 year
3	1961–1988	Wheat-corn rotation, low crop yield cultivar, ploughing, remove straw, applying chemical fertilizers and manure	2 years
4	1989–1989	Uniform tillage to set up long-term experiments	1 year
5	1990–2050	Long-term fertilization experiments	2 years

**Table 2:** Carbon input (kg ha^−1^yr^−1^) from manure and straw residue in each period used in the CENTURY model at Changping, Yangling, and Qiyang sites.

Sites	1901–1960	1961–1988	1989–1989	1990–2050 (long-term experiment)		
				NPKM	hNPKM	NPKS
Changping	500	500	–	3150	4725	1000
Yangling	500	500	–	3327	4991	1998
Qiyang	500	500	–	5838	8757	1052

Weather data from 1990–2010 were obtained from China meteorological sharing service system (http://cdc.cma.gov.cn/). For the future simulations (i.e., 2011–2050), we used monthly mean climate data from the 1990–2010 period. Soil properties parameters were soil texture (sand, silt and clay content), soil pH, and bulk density (Table 3). Soils from the Changping and Yangling sites had high soil pH (∼8.5) and silt loam texture with similar clay minerals (e.g., Hydromica and Montmorillonite). On the contrary, soil at the Qiyang site, developed from Quaternary red clay, had a lower soil pH (5.7) and heavy texture with the main clay mineral of Kaolinite.

**Table 3:** Soil classification and initial physical and chemical properties (0–20 cm) in 1989.

Properties		Changping	Yangling	Qiyang
Soil classification (FAO)		Haplic Luvisol	Calcaric Regosol	Eutric Cambisol
Parent material		Diluvial Alluvium	Loess	Quaternary Red Clay
Clay mineral type		Hydromica, Montmorillonite	Hydromica, Montmorillonite	Kaolinite
Bulk density	(g cm^−3^)	1.58	1.30	1.19
Total porosity	(%)	40.4	49.6	51.7
Field capacity	(%)	24.8	21.2	23.7
Texture		silt loam	silt loam	clay
Clay (<2 µm)	(%)	10.2	16.8	40.9
Silt (2–50 µm)	(%)	72.0	51.6	27.7
Sand (50–2000 µm)	(%)	16.2	31.6	31.4
Soil pH		8.7	8.6	5.7
SOC	(g kg^−1^)	7.1	6.3	8.6
TN	(g kg^−1^)	0.80	0.83	1.07
C/N ratio		8.9	7.6	8.0

**Figure 2:**
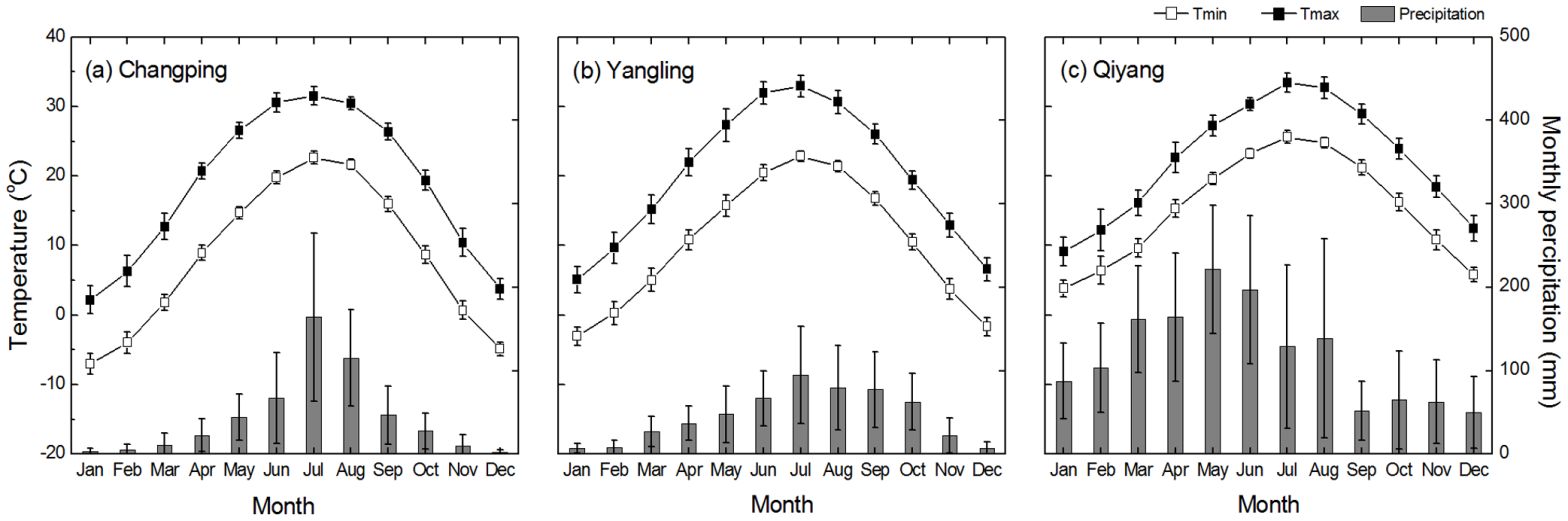
Average monthly precipitation, maximum (Tmax) and minimum (Tmin) temperatures from 1990 to 2010 at (A) Changping, (B) Yangling, and (C) Qiyang. Error bars are standard deviations for the mean values from 1990–2010.

As shown in Fig. 2, Changping and Yangling sites had similar climate condition whereas temperature and precipitation were much higher at the Qiyang site. The humid and warm climate at Qiyang site would accelerate decomposition of soil organic matter [28]. After setting up all the information (e.g., climate condition, soil texture and etc.) and modeling crop growth successfully, we modeled soil organic carbon pool by calibrating the dec4 value (i.e., maximum decomposition rate of soil organic matter with slow turnover) in the fix.100 file. By raising the parameter 10% higher for every test compared with the measured SOC, we finally got the sound fixed parameter as 0.0045 at the Qiyang site but 0.0023 (i.e., default value in the CENTURY model) at the other two sites [28].

For the plant production submodel, there were pools for live shoots and roots, and standing dead plant material. The potential production was a function of a genetic maximum (PRDX(1)) defined for each crop and 0–1 scalars depending on soil temperature, moisture status, shading by dead vegetation, and seedling growth. The maximum potential production of a crop, unlimited by temperature, moisture or nutrient stresses, was primarily determined by the level of photosynthetically active radiation, the maximum net assimilation rate of photosynthesis, the efficiency of conversion of carbohydrate into plant constituents, and the maintenance respiration rate [30]. Thus, the parameter for maximum potential production (PRDX(1)) had both genetic and environmental components. However, in CENTURY, the seasonal distribution of production was primarily controlled by the temperature response function rather than the seasonal variation in photosynthetically active radiation, so the maximum potential production parameter would reflect aboveground crop production in optimal summer conditions. This parameter would frequently be used to calibrate the predicted crop production for different environments, species, and varieties [31].

The CENTURY model had no function for pH effects on aboveground growth. Based on the 20 years of field measurement at the Qiyang site, soil pH had no impact on crop growth as long as the value remains above pH 5.5, and would completely stop growth in wheat and corn if the value declined to less than pH 4.0 [32]. We added the following soil pH factor (*f_pH_*) in the crop growth sub-model to account for this effect:
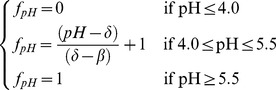
(1)where *δ* and *β* were the maximum (i.e., *δ* = 5.5) and minimum (i.e., *β* = 4.0) pH value that effected plant growth, respectively.

### Model Evaluation

We compared the simulated SOC (0–20 cm) with field measured values. Both visual examination of graphic output and several statistical tests were used to evaluate the CENTURY model performance. Four statistical parameters were selected [33]–[36] for the evaluation, including (*i*) the sample coefficients of determination (*R^2^*), (*ii*) the normalized-root mean square error (*n-RMSE*, equation 2), and (*iii*) the index of agreement (*d*, equation 3), as a descriptive measure of the average relative error [37].
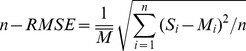
(2)where *M_i_* were the measured values, *S_i_* were the simulated values, 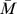
was the mean of the measured data and *n* was the number of the paired values. The index of agreement is computed with the following equation:
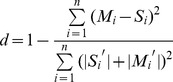
(3)


where 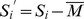
and 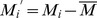.

The classical *R^2^* statistic (0<*R^2^*<1) provided the percentage of data variance that was accounted for by the model. The *RMSE* evaluated the difference between observed and modeled values in the original units of the data, while the normalized-*RMSE* (*n-RMSE*, equation 2) removed the influence of the units, and placed the results on a percentage scale for comparison of model performance among variables with different units. For an ideal fit, *R^2^* would equal 100%, *RMSE* would equal zero and *d* would equal 1. In our study, *RMSE*<15%, *d*>0.5 and significant *R^2^* were used to bound the best case parameter sets for both calibration and validation simulations [38].

## Results

### Model Evaluation

**Figure 3:**
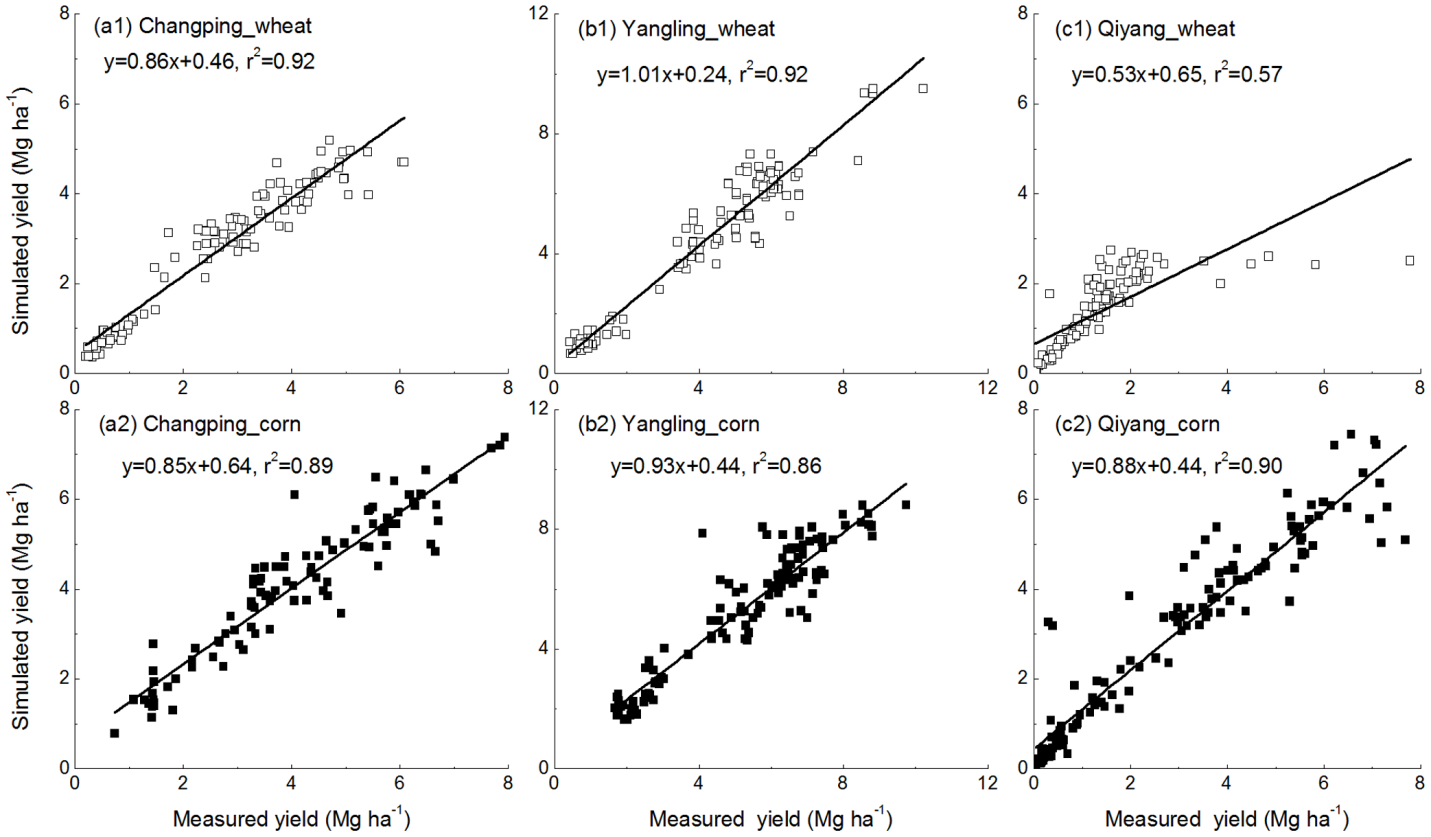
Correlationship between simulated and observed crop (i.e.,□wheat and ▪corn) grain yield data under the control, N, NP, NPK, NPKM, hNPKM and NPKS treatments at (A) Changping, (B) Yangling, and (C) Qiyang sites.

We evaluated the model results for both grain yields and the SOC stock predictions. We were focusing on the SOC stock predictions of the model. In summary for the grain yield results (Fig. 3), we found that the model had correlation coefficients (*R^2^*) ranging from 0.57 to 0.92 (*p*<0.05), indicating that CENTURY model simulates the grain yields with reasonable accuracy.

**Figure 4:**
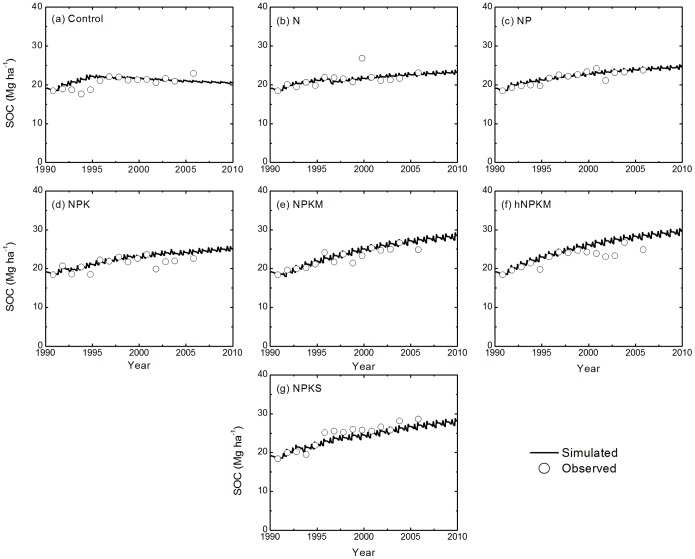
Simulated and measured soil organic carbon (SOC) stocks (0–20 cm) under the control, N, NP, NPK, NPKM, hNPKM and NPKS treatments at Changping.

**Figure 5:**
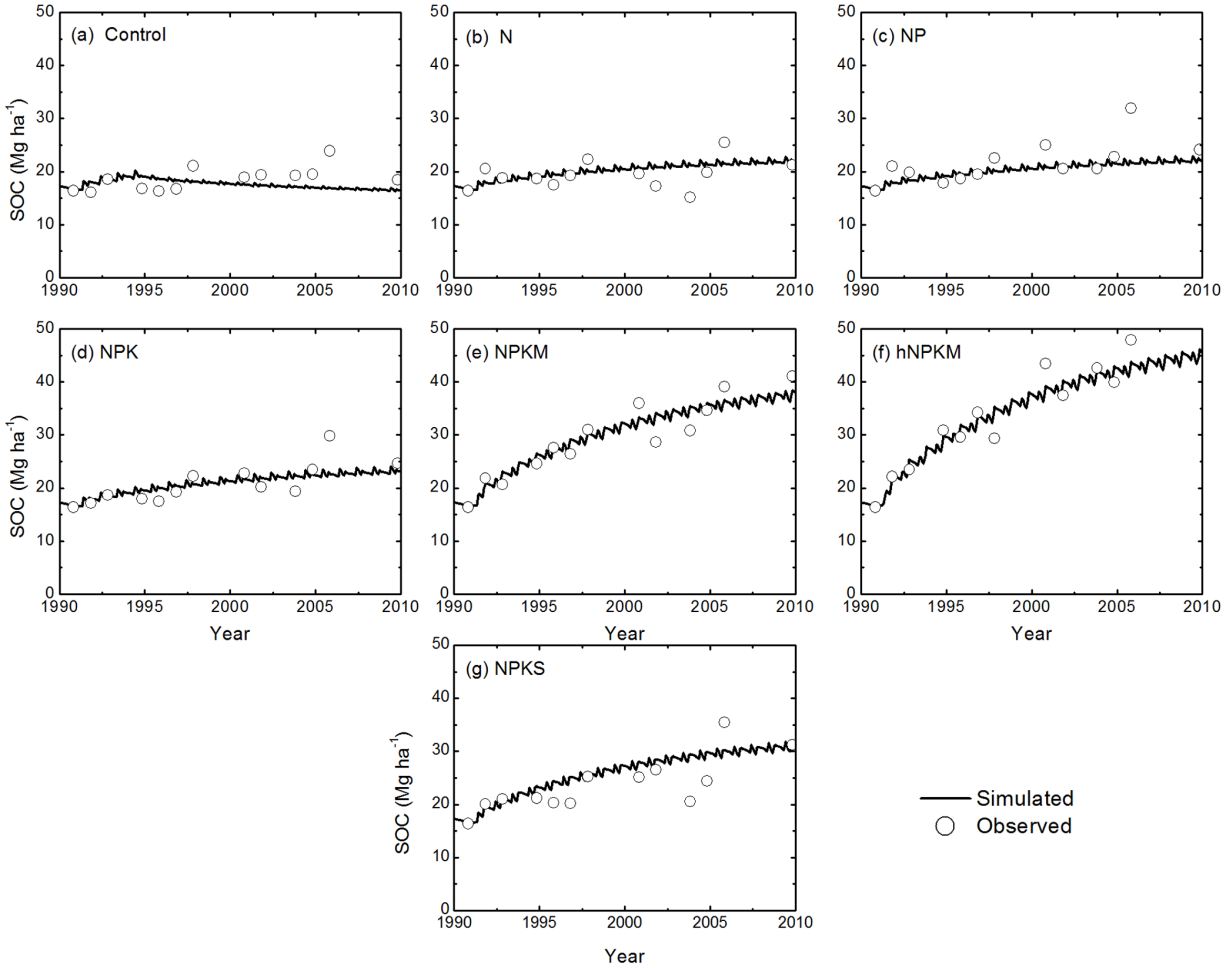
Simulated and measured soil organic carbon (SOC) stocks (0–20 cm) under the control, N, NP, NPK, NPKM, hNPKM and NPKS treatments at Yangling.

**Figure 6:**
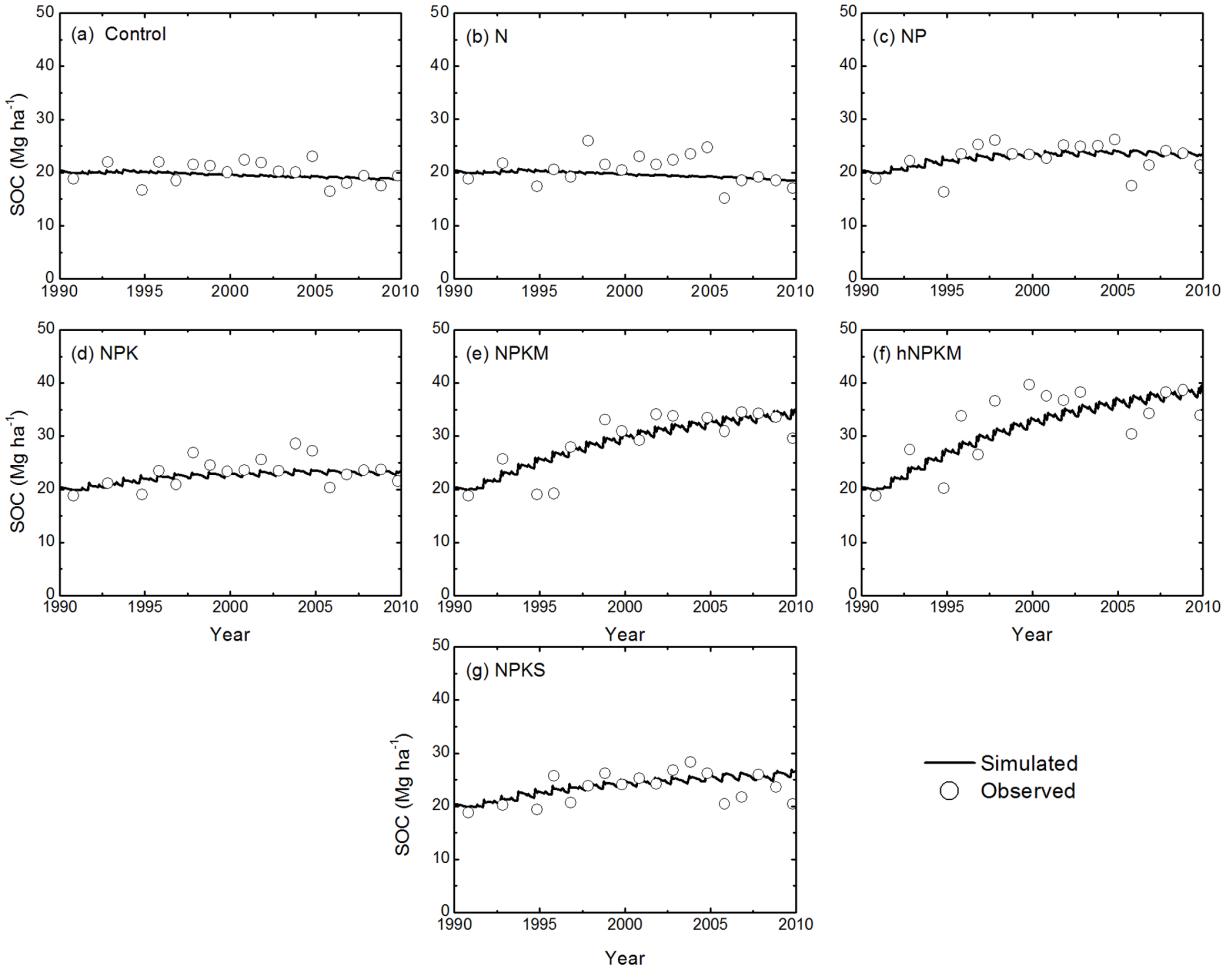
Simulated and measured soil organic carbon (SOC) stocks (0–20 cm) under the control, N, NP, NPK, NPKM, hNPKM and NPKS treatments at Qiyang.

The modeled SOC follows the trend of the measurements reasonably well in all treatments across the sites (Figs. 4, 5, and 6). Model results and measured SOC demonstrated that carbon levels decrease in the control treatment at all sites but increase under the fertilization treatments, except N only. For the N treatment, SOC is maintained at a stable level at most sites but did decline at the Qiyang site. The reason was related to a low soil pH that caused a decline in crop production and thus decline in residue input to the soil [25].

**Figure 7:**
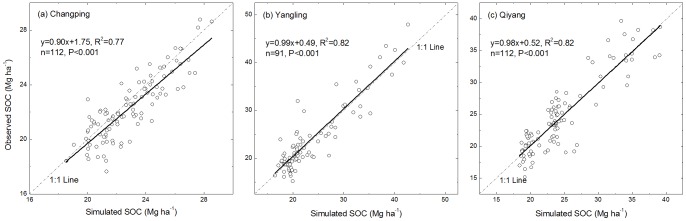
Correlation between simulated SOC and measured SOC under the control, N, NP, NPK, NPKM, hNPKM and NPKS treatments at (A) Changping, (B) Yangling, and (C) Qiyang sites.

Linear regressions of observed vs. simulated SOC stocks (Fig. 7) are all highly significant (*P*<0.001) under various fertilization treatments. Approximately 77%–84% of the variability in the observed SOC stocks can be explained by the simulation. Therefore, in our experiment, the CENTURY model performs well and is suitable for predicting SOC dynamics for different types of N fertilizer (i.e., manure *versus* mineral) and N rates. The coefficients of determination (*R^2^*) are higher at the Yangling and Qiyang sites than the Changping site.

*RMSE* is useful for evaluation of precision in model results [33]. Several studies have emphasized the need to estimate the precision in model predictions to evaluate model uncertainty [14], [39], [40]. In this study, we used several parameters for quantitative statistical analysis of measured and simulated SOC (Table 4). The *n-RMSE* ranges from 4% to 7% at the Changping site, 9%–14% at the Yangling site, and 6%–11% at the Qiyang site, respectively. Similarly, Fallon and Smith [41] calculated *n-RMSE* ranging from 1.8% to 16.4% when they modeled SOC in several long-term experiments in Europe. Also Álvaro et al. [20] had a low *n-RMSE* (below 6%) when they used the CENTURY model to evaluate tillage effects on SOC. Overall, the *n-RMSE* value is less than 15% for all the treatments in our study, demonstrating that the model is relatively precise in predicting the trends for different fertilization practices that are common in Chinese agricultural systems.

**Table 4:** Statistics comparing simulated and measured SOC stocks at the three long-term experimental sites.

Site	Parameter^a^	Treatment						
		Control	N	NP	NPK	NPKM	hNPKM	NPKS
Changping	*n-RMSE*	6%	5%	5%	6%	7%	4%	6%
(n = 16×7)	*d*	0.57	0.81	0.9	0.81	0.91	0.97	0.91
	*R^2^*	0.01	0.76	0.83	0.46	0.80	0.90	0.76
Yangling	*n-RMSE*	9%	12%	9%	12%	9%	9%	14%
(n = 13×7)	*d*	0.5	1.00	0.83	0.77	0.96	0.97	0.85
	*R^2^*	0.39	0.19	0.43	0.59	0.89	0.88	0.61
Qiyang	*n-RMSE*	8%	7%	6%	9%	10%	11%	7%
(n = 16×7)	*d*	0.37	0.51	0.75	0.61	0.89	0.88	0.82
	*R^2^*	0.07	0.16	0.47	0.34	0.69	0.64	0.61

a Note: *n-RMSE*, the normalized-root mean square err; *d*, the index of agreement; *R^2^*, the sample correlation coefficient.

The *d* values and coefficients of determination (*R^2^*) are highest in the NPKM, hNPKM and NPKS treatments at most sites. However, the *R^2^* value was much lower in the control (∼0.07) or N (∼0.19) treatments. The reason was that inter-annual fluctuations in crop yield were larger in the control and N treatments due to the long-term nutrient deficiency [25]. Therefore, carbon input from biomass was fluctuated and thus the simulation was not satisfied as other treatments.

### Fertilization Effects on Organic Carbon Pools

**Figure 8:**
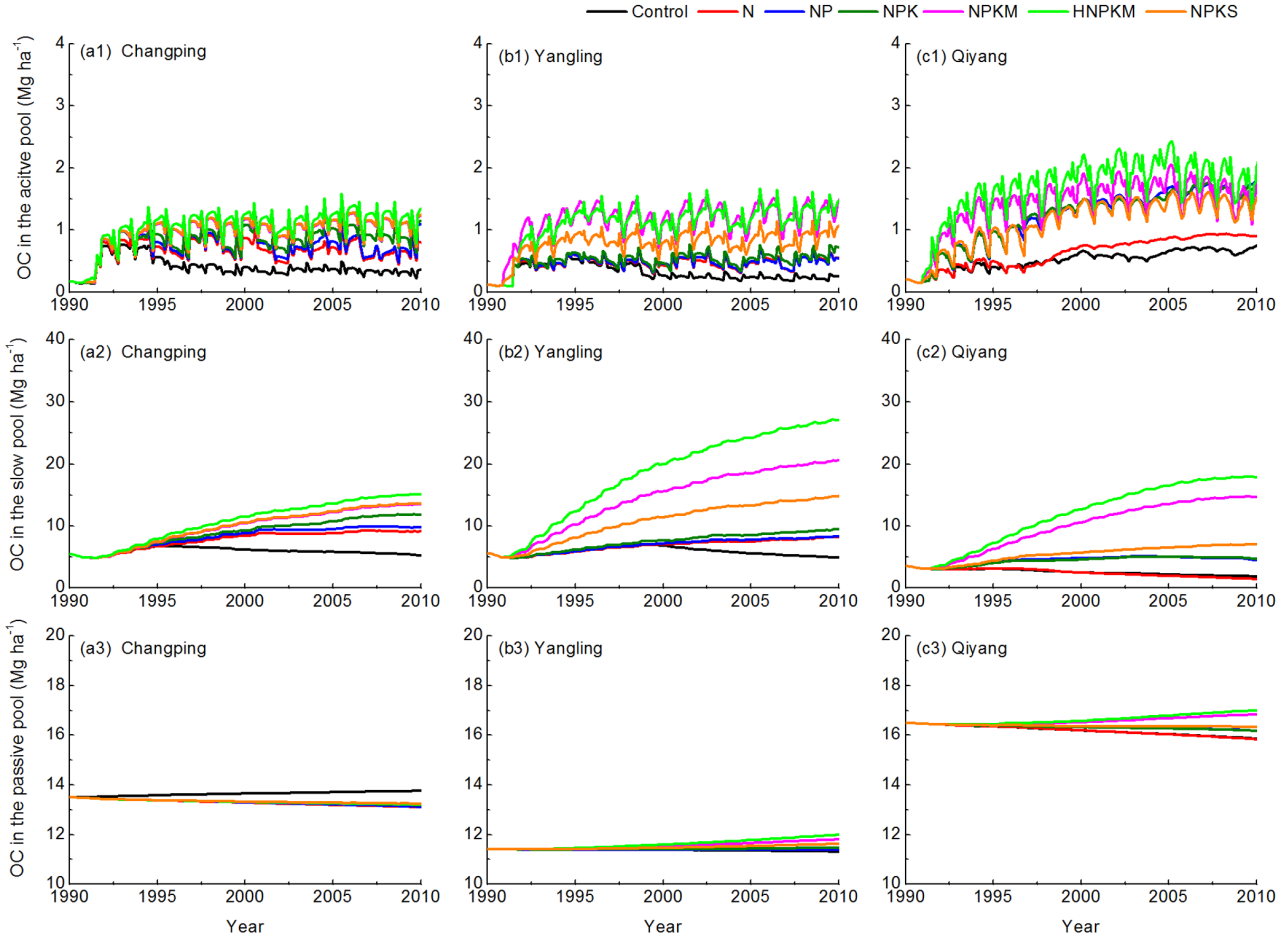
Soil organic carbon in the (1) active, (2) slow, and (3) passive pool under the control, N, NP, NPK, NPKM, hNPKM and NPKS treatments at (A) Changping, (B) Yangling, and (C) Qiyang sites.

**Figure 9:**
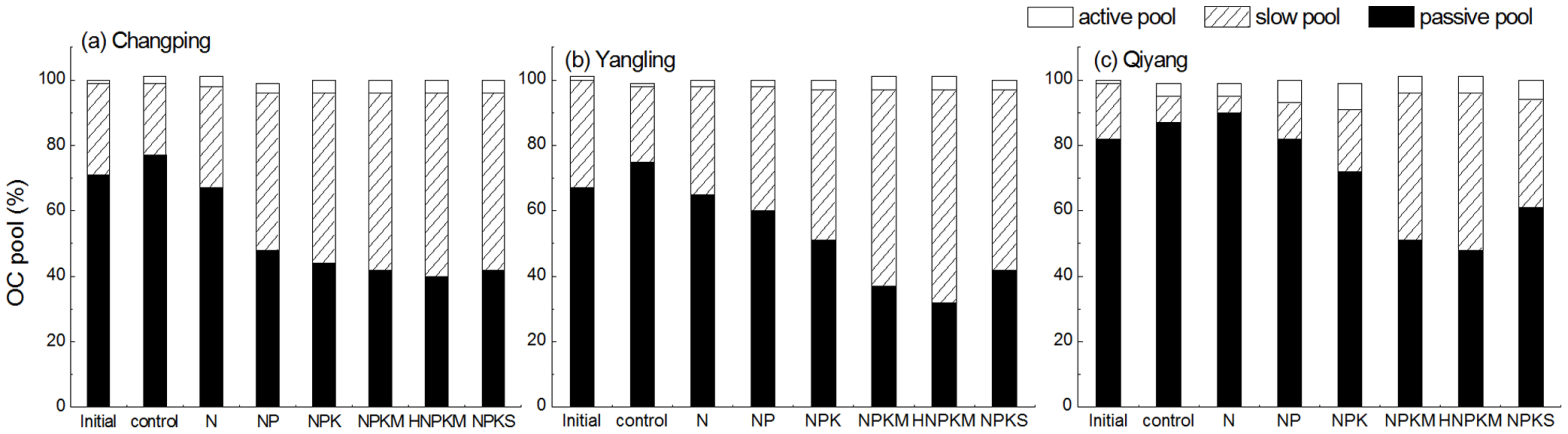
Proportion of changed SOC between the 1990 (initial) and 2010 in each soil organic matter pool under the control, N, NP, NPK, NPKM, hNPKM and NPKS treatments at (A) Changping, (B) Yangling, and (C) Qiyang sites.

Long-term fertilization has a variety of effects on SOC pools (Fig. 8 and 9). Generally, the active pool is the smallest carbon pool [16], and our results are consistent with past studies with an active pool containing only 1–3 Mg ha^−1^ across the sites (first row in Fig. 8). By 2010, the largest active pool is at the Qiyang site (Fig. 8_c1), followed by Changping (Fig. 7_a1) and Yangling (Fig. 8_b1) sites.

Long-term manure fertilization significantly increased the proportion of slow soil organic matter at all sites (Fig. 8). By the end of 2010, the slow pool accounted for ∼42% of the total SOC at the Changping site, ∼65% at the Yangling site, and ∼48% at the Qiyang site (Fig. 9). In addition, trends in the slow pool are consistent with the overall change in total SOC stock at each of the sites (Figs. 4, 5, and 6). These results suggest that the carbon dynamics associated with the slow pool are key drivers of the trend in total SOC.

The percentage of SOC in the passive soil organic matter is 75%–87% under the control treatment but only 32%–48% under the hNPKM treatment (Fig. 9). Our modeling results suggest that a higher proportion of the organic carbon is contained in the passive pool with the slowest turnover rate if no fertilizer is added. However, long-term fertilization generally has a smaller effect on the passive pool compared to the slow pool according to the experimental data (third row in Fig. 8). The reason would be corresponded to turnover times of 25 years for the slow pool but 1000 years for the passive pool in the CENTURY [28].

### Soil Carbon Dynamics and Carbon Sequestration Potential

**Figure 10:**
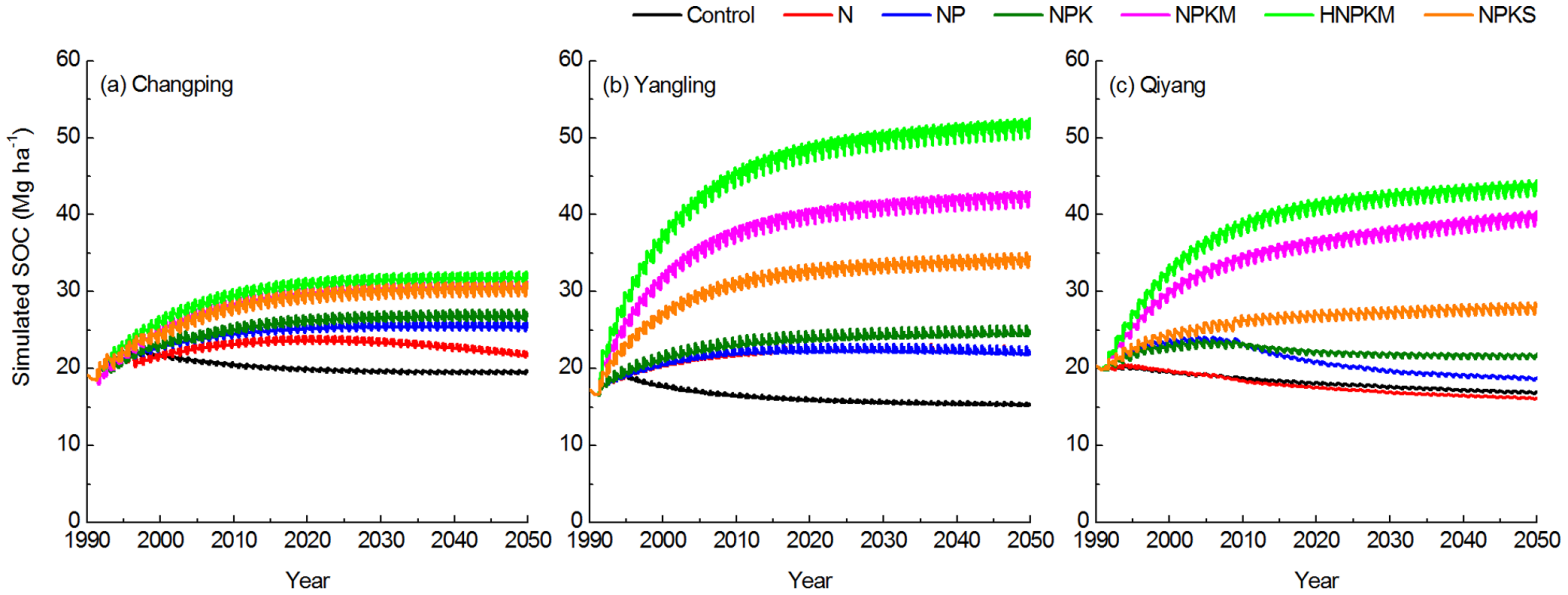
Soil organic carbon dynamics under the control, N, NP, NPK, NPKM, hNPKM and NPKS treatments at (A) Changping, (B) Yangling, and (C) Qiyang sites.

Long-term fertilization has different effects on soil organic carbon across the three sites (Fig. 10). For example, the SOC stock (0–20 cm) increases and then decreases to a relative stable level in the control and N treatments at Changping site (Fig. 10a). Plots with balanced fertilization or manure application and straw return (i.e., NPK, NPKM, hNPKM and NPKS) have an increase in SOC and then remain relatively stable. By 2050, the SOC stocks reach 30.5–31.8 Mg ha^−1^ with organic fertilization and 26.8 Mg ha^−1^ with chemical fertilization (NPK).

For the Yangling site (Fig. 10b), SOC contents increase over time in the NPK, NPKM, hNPKM, and NPKS treatments. By the end of simulated period, SOC reaches 42.6 Mg ha^−1^ and 52.1 Mg ha^−1^ under the NPKM and hNPKM treatments, respectively. With straw return, the SOC level reaches 34.3 Mg ha^−1^. Balanced chemical fertilization (NPK) also yields relatively high SOC contents at 24.6 Mg ha^−1^ by 2050. There is little difference in SOC stocks between N and NP treatments. SOC increases to 20.1 Mg ha^−1^ in 1994, then declines to 15.3 Mg ha^−1^ in 2050 for the control treatment.

Similar to the Yangling site, SOC levels increase through the entire period of simulation under the NPKM, hNPKM, and NPKS treatments for the Qiyang site (Fig. 10c). By 2050, SOC stocks are 40.2 Mg ha^−1^, 44.2 Mg ha^−1^, and 28.2 Mg ha^−1^ for the NPKM, hNPKM, and NPKS treatments, respectively. For all chemical fertilization treatments, we assume no decrease in pH after year 2010. Therefore, soil pH has not negative impact on carbon input due to a reduction in crop production associated with pH, and applying NPK fertilizers maintain SOC levels at 21.7 Mg ha^−1^.

The SOC trends are relatively stable by the end of 2050 in the different treatments (Fig. 10). In order to estimating the carbon sequestration potential (i.e., the SOC increment by the end of simulation) with manure addition and regular straw incorporation, we further evaluated the SOC change under the hNPKM and NPKS treatments in the period of 1990–2050 (Table 5). The initial SOC level is highest at the Changping site (22.4 Mg ha^−1^) and lowest at the Yangling site (16.4 Mg ha^−1^). In contrasts, the highest simulated SOC level in 2050 happens at the Yangling site and lowest at the Changping site. Compared with the NPKS treatment, carbon sequestration potential is greater with hNPKM management, ranging from 9.4 Mg ha^−1^ to 35.7 Mg ha^−1^. For the NPKS treatment, SOC increases to 17.9 Mg ha^−1^ at the Yangling site but only 7.1 Mg ha^−1^–8.1 Mg ha^−1^ at the other two sites.

**Table 5:** Estimated carbon sequestration potential (0–20 cm) under long-term hNPKM and NPKS fertilization treatments.

Site	SOC in 1990 (Mg ha^−1^)	SOC in 2050 (Mg ha^−1^)		Carbon sequestration potential (Mg ha^−1^)	
		NPKS	hNPKM	NPKS	hNPKM
Changping	22.4	30.5	31.8	8.1	9.4
Yangling	16.4	34.3	52.1	17.9	35.7
Qiyang	20.5	28.2	44.2	7.7	23.7

## Discussion

Studies has proved that optimizing fertilization managements can raise crop yield as well as biomass, which would enhance biomass input into the soil from crop straw and roots [28], [42], [43]. Halvorson et al [44] reported that increasing nitrogen fertilizer rate would increase SOC levels in the 0–7.5 cm soil depth after 11 years cropping. In our study, chemical fertilization (expect N treatment) would also increase crop yield and thus increase SOC in the first 20 years of crop rotation at two of three sites. However, most chemical fertilization treatment would only maintain the SOC level in the long-term run (Fig. 10).

Straw incorporation would also increase carbon input to the soil. Li et al [45] found that cropland soil lost 1.6% of SOC due the less (25%) aboveground residue’s returning. Using the DNDC model, Tang et al. [46] predicted that the soil carbon loss in China’s cropland would be partly reversed if straw return and no tillage practices were expanded widely. In our study, averaged carbon sequestration rate would reach to 118 kg ha^−1^ yr^−1^ to 298 kg ha^−1^ yr^−1^ with straw returning during the period of 1990–2050 under current returning rate of straw residue (i.e., 2000–4500 kg ha^−1^ yr^−1^). The results are lower than the estimated rate (108–728 kg ha^−1^ yr^−1^) in the similar region by Lu et al [47], in which the estimated straw returning rates were also higher (4530–8110 kg ha^−1^ yr^−1^).

Moreover, there are differences in the influence of manure on SOC trends among the sites. At the Changping site, SOC stocks reach a relatively stable level (30.0 Mg ha^−1^–31.0 Mg ha^−1^) by 2013 (Fig. 10a). In contrast, plots from the other two sites continue to increase in SOC over the entire simulated period. The reason for the site difference in the simulated trends is related to the manure quality between the sites, and the associated decomposition rates of the organic matter [28], [48]. For the Changping site, farmyard manure is livestock manure mixed with soil and/or crop residue. The lignin content in farmyard manure is lower and decompose at a faster rate [49]. When we enhanced the lignin content of farmyard manure in CENTURY, the simulation of SOC rose to the much higher level (data not shown).

Many modeling research also focus on the regional estimation of SOC sequestration. Follett [50] estimated soil carbon sequestration potential would be 11–56 Tg C yr^−1^ for residue management in the USA. In China, Yan et al [51] estimated 32.5 Tg C yr^−1^ to be sequestered in croplands if 50% of the crop residue was returned to soils, and no tillage was adopted on 50% of the arable lands. However, these results are criticized for the models that are calibrated against few or no field experimental data. Our study estimated carbon sequestration potential using long-term experiment data, providing credible data to evaluate carbon sequestration response to different fertilization managements.

## Conclusion

We used the CENTURY model (version 4.5) to simulate soil organic carbon (SOC) dynamics for long-term fertilization experiments with wheat-corn cropping systems in China. Our study indicated that CENTURY can simulate fertilization effects on SOC trends for different climatic conditions and soil properties that are common for wheat-corn systems in China. For most sites, the model was more precise in predicting the SOC trends for treatments with balanced nutrient additions (NPK, NPKM, hNPKM, and NPKS) than the control and unbalanced fertilization treatments (N and NP). After simulating SOC dynamics from 1990–2050, the SOC levels increased from 31.8–52.1 Mg ha^−1^ across the four sites. We estimate that carbon sequestration potential ranges from 9.4–35.7 Mg ha^−1^ under the high manure application practice (i.e., hNPKM treatment). Analysis of the proportion of organic carbon in each of the soil organic matter pools indicates that long-term fertilization enhances the slow pool and leads to a decline in the proportion of carbon in the passive pool. Model results suggest that changes in slow organic matter drive the overall trends in SOC stocks associated with long-term fertilization.
